# Basic Psychological Needs in the Work Context: A Systematic Literature Review of Diary Studies

**DOI:** 10.3389/fpsyg.2021.698526

**Published:** 2021-10-18

**Authors:** Lynelle Coxen, Leoni van der Vaart, Anja Van den Broeck, Sebastiaan Rothmann

**Affiliations:** ^1^Optentia Research Unit, North-West University, Vanderbijlpark, South Africa; ^2^Faculty of Economic and Management Sciences, School of Industrial Psychology and Human Resource Management, North-West University, Vanderbijlpark, South Africa; ^3^Department of Work and Organisation Studies, Faculty of Economics and Business, KU Leuven, Leuven, Belgium

**Keywords:** basic psychological needs, autonomy, competence, relatedness, systematic review, self-determination theory, diary studies

## Abstract

According to the self-determination theory, individuals' basic psychological needs for autonomy, competence, and relatedness should be satisfied for optimal psychological growth. The satisfaction of these needs seems to vary due to changes in a person's social context, and the outcomes of the satisfaction of these needs also vary along with the needs. Despite several studies investigating daily and weekly variations in need satisfaction and its correlates, no systematic investigation exists. This study aimed to conduct a narrative synthesis of existing quantitative diary studies of basic psychological needs in the work context. We specifically aimed to evaluate if psychological need satisfaction varies daily and weekly and judge whether they vary more daily or weekly. Additionally, we also aimed to review the literature regarding the relations between daily or weekly variations in need satisfaction and its assumed antecedents and outcomes. We included peer-reviewed articles in English that measured work-related basic psychological needs using a quantitative diary study design. Database searching (Web of Science, ScienceDirect, EBSCOhost, and Scopus) led to the extraction of 2 251 records by February 2020. Duplicates were removed, the remaining records were screened (*n* = 820), and 30 articles were assessed using eligibility criteria. Two authors individually conducted the screening and eligibility processes to manage selection bias. In total, 21 articles were included in the final review. The review indicated that basic psychological need satisfaction showed considerable within-person variation and was more dynamic daily (compared to weekly). Job demands, job resources, organisational resources, and individual characteristics appeared to associate with these variations. The organisational context seemed to matter the most for need satisfaction. Variations in need satisfaction were also related to employee well-being, performance, and motivation. Despite the small number of published studies (particularly for weekly studies), our results indicate that researchers should pay attention to within-person variations in need satisfaction. Measuring daily need satisfaction could be prioritised. Different antecedents and outcomes seem to be associated with different needs. Thus, when needs are viewed as distinct constructs instead of unidimensional ones, one can derive greater insights. The study is funded by the National Research Foundation.

## Introduction

From a self-determination theory (SDT) perspective, the satisfaction of basic psychological needs is essential for autonomous motivation, well-being, and work performance (Deci et al., 2017; Ryan and Deci, 2019). Three needs are considered essential. These are the needs for autonomy (i.e., the need to make free choices), competence (i.e., the need to master tasks), and relatedness (i.e., the need to connect with others) (Deci and Ryan, 2000).

Within the research domain, basic psychological need satisfaction has received extensive attention in the work context (Ryan and Deci, 2017). Studies have shown how individuals' need satisfaction differ from one another (i.e., between-person). Considering that the satisfaction of these psychological needs is dependent on changes in the environment (Deci and Ryan, 2000) and that the environment and perceptions thereof are not static, it is plausible that the experience of need satisfaction fluctuates within persons over time (Ryan and Deci, 2017). Therefore, scientific information is needed regarding how the same individuals' experiences of need satisfaction fluctuate (i.e., within-person) over time. Understanding these intra-individual fluctuations is essential, as it brings us closer to understanding real-life, naturally occurring phenomena (Bolger et al., 2003).

Intensive research designs, such as diary studies, enable researchers to capture within-person changes (Bolger et al., 2003; Sonnentag and Geurts, 2009). Diary studies may help facilitate an understanding of not only the degree to which needs vary but also with which individual (e.g., personality) and contextual (e.g., job characteristics) antecedents and outcomes (e.g., well-being and performance) (Bidee et al., 2017) such variations correlate. Within-person studies are also valuable from a methodological point of view. Apart from gathering real-life data (contributing to high external validity) (Bolger and Laurenceau, 2013), recall bias is minimised (Bolger et al., 2003; Sonnentag and Geurts, 2009).

The work context is an area in which diary studies are increasing. Some researchers tapped into within-person variations of psychological need satisfaction at work (e.g., Aldrup et al., 2017; Bakker and Oerlemans, 2019; Van Hooff and De Pater, 2019). These diary studies have either taken a daily (e.g., Haar et al., 2018) or a weekly (e.g., Weigelt et al., 2019) approach to study variations in psychological needs. Although Van den Broeck et al. (2016) advocated for diary studies on basic psychological needs in the work context and some researchers heeded this call, we still lack a systematic understanding of whether the needs fluctuate and how often, and which factors are associated with this variation. Without a summary of current evidence of fluctuating need satisfaction in the work context, contributions to evidence-based practises and future research avenues remain limited. From this summary, we might assess whether the use of daily or weekly studies would be most beneficial. Therefore, a review could guide future studies on whether a within-person approach would be more suitable and on which level(s) (i.e., daily or weekly) need satisfaction should be studied.

Cross-sectional studies on psychological need satisfaction and its relations with potential antecedent and outcome variables have been synthesised (see Van den Broeck et al., 2016). However, it would be valuable to determine if variations in need satisfaction relate to variations in antecedents and outcomes. Thus, reviewing need satisfaction's relationships with other variables (e.g., potential antecedents and outcomes) could facilitate an understanding of the factors that might affect within-person variations in need satisfaction and the consequences (i.e., outcomes) thereof. Furthermore, a review could facilitate an understanding of which antecedents and outcomes of fluctuations in need satisfaction are already well-studied and which areas require more research.

This study aimed to review diary studies on work-related basic psychological need satisfaction. Specifically, this study aimed to (1) evaluate if need satisfaction varies on a daily and weekly level, and to judge on which level the needs vary more, and (2) to examine the associations between fluctuating need satisfaction and its associated categorised antecedents and outcomes.

In accomplishing these aims, the review contributes to SDT literature by synthesising the available diary study literature on basic psychological need satisfaction in the work domain and providing directions for future research studies utilising a diary method design. Practically, this study can help organisations design and plan interventions that may contribute to high within-person levels of need satisfaction and positive employee and organisational outcomes. Practitioners may use the review to help them create an environment that promotes satisfaction of basic needs both between and within persons.

## Literature Overview

### Basic Psychological Needs

SDT is a theory of human motivation (Deci et al., 2017) that examines how social or contextual factors can either enhance or inhibit people's experiences of the satisfaction of three basic psychological needs (Deci and Ryan, 2000; Van den Broeck et al., 2016). The need for *autonomy* refers to “volition” and willingness and is concerned with people's aspiration to self-organise their experiences to ensure that activities are consistent with their sense of self (Deci and Ryan, 2000). It is satisfied once a person can make choices freely and, subsequently, experience ownership of their behaviour (Deci and Ryan, 2000). *Competence* satisfaction refers to the experience of mastery and effectiveness when engaging in tasks (Deci and Ryan, 2000). The need for competence is fulfilled when people can perform tasks confidently and develop new skills to enable mastery in the future (Van den Broeck et al., 2016). Finally, *relatedness* satisfaction refers to a person's desire to experience warm, meaningful, and close connexions with significant others (Deci and Ryan, 2000). The need for relatedness is satisfied when people experience a sense of affiliation with others and develop close relationships (Van den Broeck et al., 2016).

According to the meta-analysis of Van den Broeck et al. (2016), in which literature on need satisfaction was summarised, most studies investigating basic psychological need satisfaction employed cross-sectional survey designs. Yet, some scholars are starting to adopt within-person diary study methods.

According to Bolger and Laurenceau (2013), psychological constructs should be studied as naturally developing or evolving processes. Basic psychological need satisfaction is a psychological construct that depends on the social environment and how it is perceived (Ryan and Deci, 2017). Therefore, it can be argued that need satisfaction is likely to fluctuate along with changes in the environment or perception thereof. Recent diary studies showed that basic psychological needs might fluctuate daily (Bidee et al., 2017) and weekly (Petrou and Bakker, 2016). To provide more systematic insights into these fluctuations and whether they occur, we aim to focus on the following review objective:

*Review Objective 1:* To investigate whether basic psychological need satisfaction varies at the within-person level on a daily and weekly basis and to judge whether the needs vary more on a daily or weekly level.

If basic need satisfaction is likely to vary, it is essential to understand its associations with antecedents and outcomes. Building on the meta-analysis of Van den Broeck et al. (2016), we aim to gain a comprehensive understanding of the dynamic processes of need satisfaction by systematically reviewing the available diary studies. Similar to previous research, we (1) clustered key variables (e.g., workload and well-being) into potential antecedents and outcomes of psychological need satisfaction, and (2) categorised antecedents and outcomes into sub-categories (e.g., work environment and employee factors, employee attitudes and well-being, and behavioural and motivational outcomes, respectively).

### Cross-Sectional “Antecedents” of Basic Psychological Need Satisfaction in the Work Context

#### Work Environment

Basic psychological needs are context-responsive constructs (Vansteenkiste et al., 2020). Hence, their satisfaction depends on the organisational context in which employees operate (Vansteenkiste and Ryan, 2013; Ryan and Deci, 2017). Several cross-sectional studies investigated workplace factors as “antecedents” of need satisfaction. These factors can be categorised as job demands and resources in the job demands-resources (JD-R) model (Demerouti et al., 2001) or factors in the organisational context (referred to as organisational resources) (Van den Broeck et al., 2016). Job demands are the organisational, physical, psychological, and social job aspects that require persistent effort and may result in adverse outcomes (Demerouti et al., 2001). Job demands (e.g., high workload, work-home interference, role conflict, and role ambiguity) are generally detrimental to need satisfaction (Van den Broeck et al., 2008, 2016), but this relationship may not be straightforward. The appraisal of these demands—as challenges or hindrances—determines whether their effect is detrimental or beneficial (Crawford et al., 2010; Van den Broeck et al., 2010). Hindrances are regarded as “health-impairing job demands” that thwart optimal functioning. At the same time, challenges are seen as job demands that are motivating yet require some energy (Van den Broeck et al., 2010, p. 736). In line with this view, meta-analytic findings indicate that whereas hindrance demands (e.g., role conflict) undermine need satisfaction, challenge demands (e.g., cognitive demands) enhance need satisfaction (Van den Broeck et al., 2016). Job resources are the organisational, physical, psychological, and social job aspects resulting in goal achievement, growth, development, and the buffering of demands and costs associated with demands (Demerouti et al., 2001). In their meta-analysis, Van den Broeck et al. (2016) found that the basic needs showed significant positive relations with all the job resources they measured (e.g., autonomy, social support, and skill utilisation).

Organisations consist of multiple levels, and therefore resources manifest on five different levels: individual, group, leader, organisational, and the broader (outer) context (IGLOO framework) (Nielsen et al., 2018). Building on this framework, in this study, organisational resources refer to aspects in the organisational context that manifest on the level of the organisation (i.e., organisational support or policies) or leader (i.e., leadership behaviour). Studies found that positive leadership (e.g., need-supportive leaders, transformational leadership, and servant leadership) promoted need satisfaction (Chiniara and Bentein, 2016; Van den Broeck et al., 2016; Slemp et al., 2018). On an organisational-level, organisational support and interpersonal, and organisational justice were also positively related to need satisfaction (Gillet et al., 2012; Van den Broeck et al., 2016).

In conclusion, the literature on the antecedents of need satisfaction indicates that job demands generally relate negatively to need satisfaction if perceived as a hindrance. In contrast, the opposite can be true for challenge demands. Job and organisational resources generally relate positively to basic psychological needs. Most of these studies adopted a between-person cross-sectional approach, comparing employees who experience, for example, different degrees of workload. However, the work environment is dynamic, and an employee's demands and resources can change constantly. For instance, one's workload may differ from day-to-day. If workplace factors associate with the satisfaction of the basic psychological needs cross-sectionally, it can be hypothesised that variations in these workplace factors can be associated with variations in need satisfaction. In that case, it might be that variations in these workplace factors can be related to variations in need satisfaction. Existing diary studies have demonstrated that variations of factors in the work environment are associated with variations in basic psychological need satisfaction (Aldrup et al., 2017; De Gieter et al., 2018). Therefore, this review has the following objective:

*Review Objective 2a*: To examine if variations in job demands and job and organisational resources associate with variations in psychological need satisfaction.

#### Employee Factors

Need satisfaction depends not only on the work environment but also on how employees interpret their environment. First, in the realm of SDT, such individual differences refer to *general causality orientations* (GCOs) (Deci and Ryan, 2000). Employees can interpret their environment as supportive *(autonomous orientation*), controlling (*controlled orientation*), or beyond their control (*impersonal orientation*) (Deci and Ryan, 2000). GCOs relate positively to autonomy and relatedness satisfaction (Van den Broeck et al., 2016). Second, personal resources, defined as the personal characteristics (e.g., mindfulness, self-esteem, and self-efficacy) that have an impact on how people can control and influence their environment (Xanthopoulou et al., 2013), relate positively to need satisfaction (Van den Broeck et al., 2016). Third, biographical characteristics (e.g., age and tenure) also relate positively to need satisfaction (Van den Broeck et al., 2016). Finally, employee states (i.e., cognition, affect, and behaviours that change over time due to situational factors) (Schmitt and Blum, 2020) may influence employees' need satisfaction experiences. So, how employees feel (i.e., attitudes and well-being) in the morning before work or what they strive to do (i.e., proactiveness) during their workday could relate to their need satisfaction at work. For example, proactive work behaviour is positively related to competence satisfaction (Strauss and Parker, 2014). Therefore, this review has the following objective:

*Review Objective 2b:* To examine if variations in employee factors associate with variations in psychological need satisfaction.

Apart from studying the assumed “antecedents” of need satisfaction, scholars have also invested time in examining its expected “outcomes”.

### Cross-Sectional “Outcomes” of Basic Psychological Need Satisfaction in the Work Context

#### Employee Attitudes and Well-Being

A core assumption of SDT is that need satisfaction results in optimal functioning, growth, and well-being, including positive attitudes and behaviour, and various empirical studies confirmed this assumption (Van den Broeck et al., 2016, 2019; Van Hooff and De Pater, 2019). For example, need satisfaction is positively associated with job satisfaction and affective commitment and negatively with turnover intention (Trépanier et al., 2014; Van den Broeck et al., 2016). It also relates positively to well-being (e.g., positive affect, happiness, and life satisfaction) (Gillet et al., 2012; Van den Broeck et al., 2016), whereas it relates negatively to ill-being (e.g., negative affect, strain, and burnout) (Van den Broeck et al., 2016). Previous studies found that need satisfaction as well as employee attitudes and well-being fluctuate (e.g., Van Hooff and Van Hooft, 2017). Therefore, it can be argued that variations in need satisfaction could relate to variations in attitudes and well-being.

#### Employee Behaviours and Motivation

Another assumption of SDT is that need satisfaction facilitates employee performance and motivation (Deci and Ryan, 2000). Various empirical studies also support this assumption. For example, need satisfaction is related to performance (e.g., task, creative, and proactive), job crafting behaviours, effort (Chiniara and Bentein, 2016; Van den Broeck et al., 2016), and autonomous forms of motivation (Van den Broeck et al., 2016). From the above examples, it is evident that fluctuations in the experience of need satisfaction could be associated with changes in performance and motivation.

Apart from cross-sectional studies illustrating the associations between basic psychological need satisfaction and well-being and performance, respectively, diary studies have demonstrated that variations in need satisfaction are associated with variations in well-being and performance (Vansteenkiste and Ryan, 2013; Bakker and Oerlemans, 2019; Goemaere et al., 2019b). Therefore, this review has the following objective:

*Review Objective 3*: To examine if variations in need satisfaction associate with variations in employee attitudes, well-being, performance behaviours, and motivation.

## Methods

The literature overview on the available cross-sectional literature provided a preliminary understanding of the potential antecedents and outcomes of the basic psychological needs. The focus of this systematic review was on quantitative diary studies pertaining to basic psychological needs literature. The eight-step process for conducting systematic reviews described by Uman (2011) was followed: defining the review objectives, formulating the search strategy, determining the inclusion and exclusion criteria, screening the articles, conducting a quality assessment, extracting the data, analysing the data, and reporting the findings. Accordingly, a systematic approach was followed to select and critically appraise the available diary studies focused on work-related basic psychological needs. The 2020 version of the “Preferred Reporting Items for Systematic Review and Meta-Analysis Protocols” (PRISMA-P) reporting guidelines were used (Page et al., 2020).

### Search Strategy

A systematic search was independently conducted by the first and second authors between January and February 2020. Articles were accessed through the following databases: (a) Web of Science, (b) ScienceDirect, (c) EBSCOhost, and (d) Scopus. The only restrictions that were placed on the searches were language (i.e., English) and document type (i.e., journal articles). Searches were conducted using the Boolean search method. Three search term combinations were used in each of the databases, which yielded a total of 2,251 records that were imported into EndNote. Table 1 provides these search term combinations as well as the sample sizes.

**Table 1:** Search term combinations and sample sizes.

**Database**	**Search term 1**	**Search term 2**	**Search term 3**
Web of science	(Basic psychological need^*^) AND (diary^*^ OR daily OR weekly) (*n* = 135)	(Psychological need^*^+ self determin^*^) AND (diary^*^ OR daily OR weekly) (*n* = 203)	(Psychological need^*^+ self determin^*^) AND (diary^*^ OR daily OR weekly) AND TS = (work^*^) (*n* = 55)
ScienceDirect	“Basic psychological need” AND (“diary” OR “daily” OR “weekly”) (*n* = 140)	“Psychological need” AND “self determination” AND (“diary” OR “daily” OR “weekly”) (*n* = 261)	“Psychological need” AND “self determination” AND (“diary” OR “daily” OR “weekly”) AND “work” (*n* = 233)
EBSCOhost (Automatically removes duplicates from other searchers in EBSCOhost)	“Basic psychological need^*^” AND diary “Basic psychological need^*^” AND daily “Basic psychological need^*^” AND weekly (*n* = 120)	“Psychological need^*^” AND “self determin^*^” AND diary “Psychological need^*^” AND “self determin^*^” AND daily “Psychological need^*^” AND “self determin^*^” AND weekly (*n* = 380)	“Psychological need^*^” AND “self determin^*^” AND diary AND work^*^ “Psychological need^*^” AND “self determin^*^” AND daily AND work^*^ “Psychological need^*^” AND “self determin^*^” AND weekly AND work^*^ (*n* = 31)
Scopus	“Basic psychological need^*^” AND diary “Basic psychological need^*^” AND daily “Basic psychological need^*^” AND weekly (*n* = 295)	“Psychological need^*^” AND “self determin^*^” AND diary “Psychological need^*^” AND “self determin^*^” AND daily “Psychological need^*^” AND “self determin^*^” AND weekly (*n* = 380)	“Psychological need^*^” AND “self determin^*^” AND diary AND work^*^ “Psychological need^*^” AND “self determin^*^” AND daily AND work^*^ “Psychological need^*^” AND “self determin^*^” AND weekly AND work^*^ (*n* = 267)

Using EndNote, 1,431 duplicates were removed, and the remaining 820 article titles and abstracts were manually screened against the inclusion criteria. The screening process was conducted independently by the first and second authors. Studies were included if they were empirical research articles focusing on daily or weekly fluctuations in the basic psychological needs utilising a diary study design in the work context. After the screening process, 790 articles were excluded. These records were excluded as they either did not focus on basic psychological need satisfaction, were not daily or weekly diary studies, or were not conducted within the work context. The full-text versions of the 30 included articles were screened and their reference lists checked, which led to the inclusion of one additional record. The additional record could have been missed in the original search as the title and abstract did not make any reference to basic psychological needs.

### Eligibility Criteria

**Figure 1:**
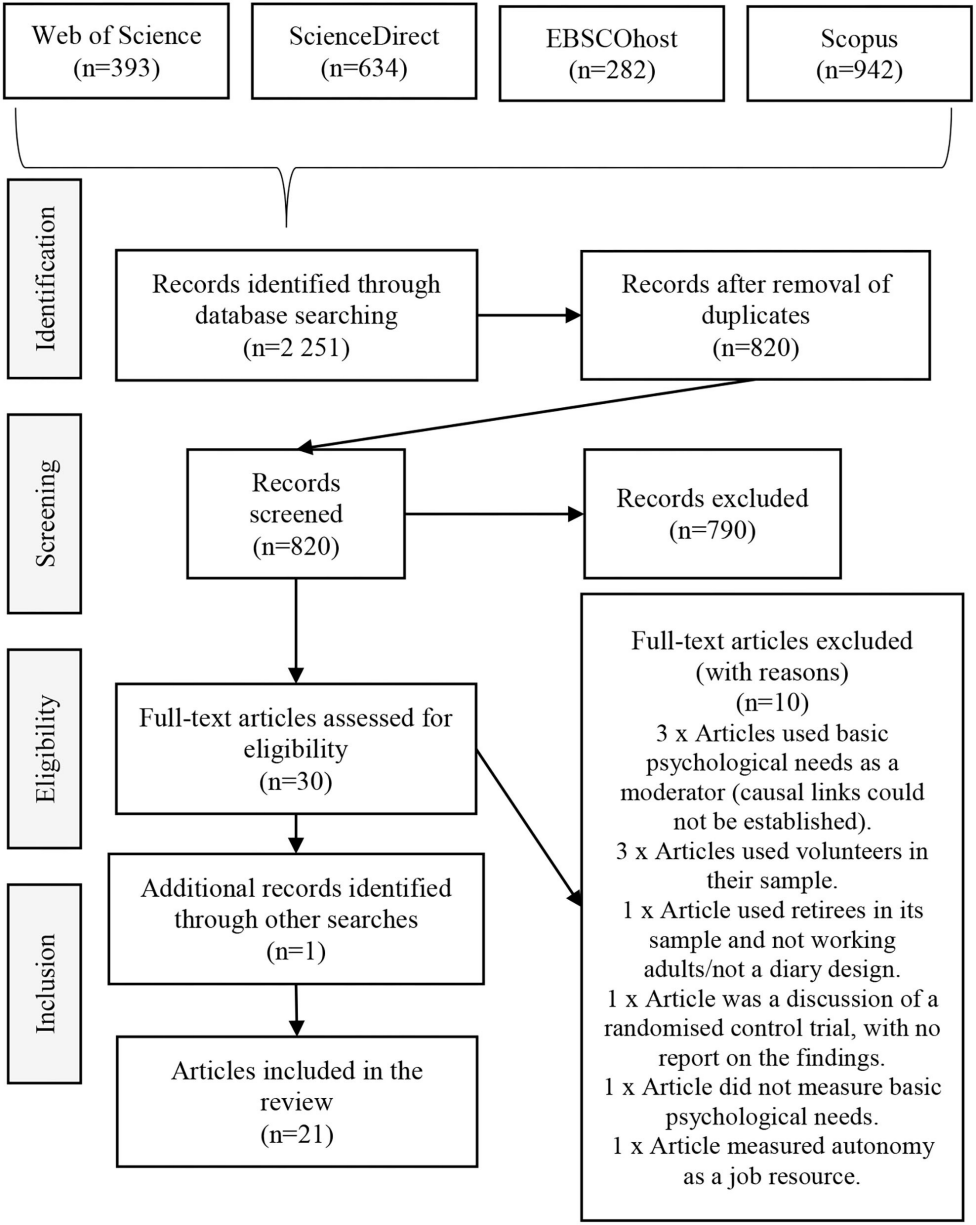
Search strategy process.

For articles to be considered as eligible for inclusion in the systematic review, the following eligibility criteria were considered for full-text article screening: (1) the sample had to be working adults; (2) basic psychological need satisfaction and/or frustration and/or autonomy; competence; and relatedness had to be studied as constructs; (3) the research approach utilised had to be quantitative diary studies; and (4) the articles had to focus on variation of the basic psychological needs and its potential antecedents and outcomes. Based on the eligibility criteria, ten articles were excluded. Figure 1 provides an overview of the complete search strategy process.

### Quality Assessment and Data Extraction

The quality of the 21 articles included was assessed using the Critical Appraisal Skills Programme (CASP), specifically the Adapted CASP for Quantitative Studies (see Laher and Hassem, 2020). Authors 1 and 2 critically appraised the included articles independently. The studies were scored out of 11, with all studies having acceptable scores (ranging between 8 and 11). Twenty-one articles were included in the review (See Table 2 for a list of the studies included). The required data was then extracted into a separate document for further processing. The extracted data consisted of the within-person variations of the basic psychological needs, their potential antecedents and outcomes, and within-person correlations. Most authors included the required information; however, the authors of some of the articles were contacted to provide additional information where needed.

**Table 2:** List of included articles.

	**Authors**	**Method**	**Autonomy**	**Competence**	**Relatedness**	**Need satisfaction**
1	Aldrup et al. (2017)	Daily diary (10 days)		√	√	
2	Bakker and Oerlemans (2016)	Daily diary (3 days)				√
3	Bakker and Oerlemans (2019)	Daily diary (4 days)	√	√	√	
4	Breevaart et al. (2020)	Weekly diary (5 weeks)				√
5	Cangiano et al. (2019)	Daily diary [5–7 days (3 times per day)]		√		
6	De Gieter et al. (2018)	Daily diary (10 days)	√	√		
7	Foulk et al. (2019)	Daily diary (10 days)				√
8	Frögéli et al. (2019)	Weekly diary (13 weeks)		√	√	
9	Goemaere et al. (2019a)	Weekly diary (8 weeks)	√			
10	Goemaere et al. (2019b)	Weekly diary (48 weeks)	√	√	√	
11	Haar et al. (2018)	Daily diary (4 days)	√	√	√	
12	Hetland et al. (2015) (Study 2)	Daily diary (5 days)				√
13	Petrou and Bakker (2016) (Study 2)	Weekly diary (3 weeks)	√	√	√	
14	Van Hooff and De Pater (2019)	Daily diary (10 days)	√	√	√	
15	Van Hooff and Geurts (2014)	Daily diary (5 days)	√	√	√	
16	Van Hooff and Geurts (2015)	Daily diary (5 days)				√
17	Van Hooff and Van Hooft (2017) (Study 2)	Daily diary (5 days)				√
18	Vandercammen et al. (2014) (Study 1)	Daily diary (10 days)	√	√	√	
19	Wang et al. (2019)	Daily diary (8 days)	√	√	√	
20	Weigelt and Syrek (2017)	Weekly diary (14 weeks)	√			
21	Weigelt et al. (2019)	Weekly diary (12 weeks)		√		

### Selection Bias

A number of strategies were employed to manage selection bias. First, Authors 1 and 2 performed the literature searches independently using the same steps and keywords and compared the search results for consistency (ensuring that no records were missed during the search process). After the independent searches, Authors 1 and 2 found the same number of articles (2,251). Second, the titles and abstracts were independently screened by the first two authors. The inter-rater reliability [Cohen's Kappa coefficient (κ)] between Authors 1 and 2 was calculated using the statistical package R (Version 4.1.0) (R Core Team, 2020). The results showed a significant agreement between the two authors, as κ exceeded the cut-off score for good inter-rater reliability of 0.80 (κ = 0.842, *p* < 0.01) (McHugh, 2012). A quality appraisal was also performed independently by the first two authors. After each phase of the search process was completed, the author and co-authors would meet to debate the inclusion and exclusion of the papers and address any agreements/disagreements.

### Certainty in Evidence

The Grading of Recommendation, Assessment, Development and Evaluation (GRADE) approach was used to assess certainty in the review results. Several factors have been assessed: (1) methodological limitations of the included studies, (2) indirectness of the results of the studies to the review's objectives, (3) imprecision of estimates, (4) inconsistency of the results, and (5) possible publication bias (Murad et al., 2017).

#### Methodological Limitations of the Studies

The included studies were assessed to be of high quality during the quality appraisal process, focusing on methodological aspects (see Adapted CASP for Quantitative Studies; Laher and Hassem, 2020). The methodologies used in the studies were relevant to the objectives of the review. Measurement points ranged between 240 and 1 619 (*n* = 6–264), which are sufficient for quantitative data analysis. Shortened questionnaires were used (a standard protocol in diary studies) to reduce participant burden/fatigue. This might mean that underlying content domains may not be fully represented. However, this is a risk in any diary study and should not influence the general results of the review.

#### Indirectness

Since the search strategy was very specific regarding sample, constructs, and method used, the primary studies were directly related to the objectives of the review. All of the included studies measured basic psychological need satisfaction, irrespective of what the research objectives were. The intraclass correlations (ICC) coefficients to evaluate if there are within-person variability in need satisfaction and judge whether the needs vary more daily or weekly (Review Objective 1) were extracted. In addition, the effect sizes of the within-person correlations in the studies were used to report whether fluctuating need satisfaction associates with antecedents and outcomes (Review Objectives 2–3). The research evidence extracted is therefore not dissimilar to the review objectives.

#### Imprecision

When all of the studies' sample sizes are aggregated, the review consisted of a sample of 2 114 employees. For Review Objective 1, the ICC-values were derived from 1,421 (daily studies) to 497 employees (weekly studies). For Review Objective 2a, 878 (daily) and 330 (weekly) employees were included, and 680 (daily) and 138 employees (weekly) for Review Objective 2b. Finally, for Review Objective 3, 1,520 (daily) and 250 (weekly) employees were included. Two of the weekly studies only had six employees over 8 and 48 weeks, respectively, which may cause problems to generalise the results of this study. Generally, the weekly studies were fewer than the daily studies. This could lower overall confidence in the certainty of weekly compared to daily diary studies' findings.

#### Inconsistency

The ICC values were relatively similar across daily as well as weekly studies. Due to the diversity of variables included in the primary studies, it was challenging to assess the similarity of the effect sizes. However, the studies that assessed similar variables' direction and magnitude of effect sizes were relatively consistent. Therefore, no major inconsistency issues were detected.

#### Publication Bias

Only peer-reviewed published studies were included in the review. Hence, we could not test for publication bias. Non-significant findings were also published, and therefore, we did not expect serious publication bias.

Based on the above GRADE analysis, the authors have a moderate to high confidence that the review findings reasonably represent the constructs/variables included.

## Results

### Variations in Basic Psychological Need Satisfaction

The intraclass correlation (ICC) coefficients reported in the included studies were used to evaluate if the basic psychological needs varied on a daily and weekly level and judge on which level the needs vary more. The ICCs reflect the proportion of the variance that lies at the between-person level, whereas 1-ICC (expressed as a percentage) reflects the within-person variance. In Table 3, the percentages used indicate 1-ICC.

**Table 3:** Intra-Individual variations in need satisfaction.

		***N***	**Minimum (%)**	**Maximum (%)**	**ME (%)**	***M*** **(%)**	***SD*** **(%)**
Daily	Autonomy	6	41.00	68.90	56.00	56.10	12.66
	Competence	8	42.00	72.70	56.00	57.57	11.40
	Relatedness	6	43.40	72.40	54.00	57.14	11.55
	Need satisfaction	5	41.00	62.60	56.40	54.60	8.66
Weekly	Autonomy	4	21.00	56.00	39.00	38.75	17.80
	Competence	4	40.00	47.00	44.00	43.75	2.99
	Relatedness	3	27.00	61.00	47.00	45.00	17.09
	Need satisfaction	1	39.60	39.60	39.63	39.63	–

*N, number of studies; ME, median; M, mean; SD, standard deviation; some of these studies overlap; Minimum and Maximum, Range. Percentages in the table indicate 1-ICC*.

The 21 studies focused on need satisfaction as either uni- or multidimensional (i.e., autonomy, competence, and relatedness satisfaction). Fourteen studies measured the needs daily (ranging between 3 and 10 days), while seven measured the needs weekly (ranging between 3 and 48 weeks). The ICC values of two of the studies could not be obtained. As displayed in Table 3, five of the 14 daily-diary studies treated need satisfaction as unidimensional and reported daily fluctuations of between 41.00 and 62.60% (ME = 56.40). One weekly study reported a variation of 39.63%. The remaining 13 studies examined basic psychological need satisfaction separately, with seven focusing on daily fluctuations and six on weekly fluctuations. Autonomy satisfaction showed a daily variation ranging between 41 and 68.90% (ME = 56.00) and a weekly variation ranging between 21 and 56% (ME = 39.00). Competence satisfaction varied between 42 and 72.70% (ME = 56.00) daily, while it fluctuated between 40 and 47% (ME = 44.00) on a weekly level. Finally, relatedness satisfaction varied between 43.40 and 72.40% (ME = 54.00) on a daily level and between 27 and 61% (ME = 47.00) on a weekly level.

Table 3 indicates that 1-ICC values range from 41.00 to 72.70% for daily need satisfaction and from 21 to 61% for need satisfaction measured at the weekly level. Therefore, it can be concluded that need satisfaction varies at the within-person level, both daily and weekly. A preliminary analysis of the mean 1-ICC values showed that the variance in daily need satisfaction could be attributed more to within- than between-person variations. The opposite seemed true for the variance in weekly need satisfaction, with more of it being attributable to between-person variations. The daily variation ranged (on average) from 54.60 to 57.57% (ME; 54.00 to 56.40%), while the weekly variation ranged between 38.75 and 45.00% (ME; 39 to 47%). Consequently, we can conclude that basic need satisfaction levels fluctuate (on average) at the within-person level, more day-to-day than from week to week. Furthermore, daily and weekly fluctuations are relatively equal across the three needs, business sectors, occupations, and countries. Therefore, basic psychological need satisfaction varies daily and weekly, with daily variations being larger than weekly variations (Review Objective 1).

### Relations Between “Antecedents” and Fluctuating Basic Psychological Need Satisfaction

In discussing the antecedent variables that appeared in the diary studies, it should be noted that almost all diary studies measured the antecedent and need satisfaction variables simultaneously at the same time of the day or week, mostly at the end of each workday or week. However, the antecedent variables were categorised based on theoretical justifications and hypotheses in the included studies.

#### Work Environment

Table 4 summarises an interpretation of the effect sizes of the within-person correlations between job demands and basic psychological need satisfaction from ten studies. All job demands, except unfinished tasks, were measured concurrently with the psychological needs, yet they are considered antecedents of need satisfaction based on theoretical grounds. The research examined job demands as diverse as emotional, physical, and cognitive demands as well as stress, work pressure, workload, job insecurity, work and family conflict, and time spent on work tasks. In general, fluctuating job demands correlated negatively with fluctuating needs, mostly with a small effect. The exceptions were time spent on core tasks, client interactions, and meetings, as they were positively related to need satisfaction. Associations between autonomy and relatedness satisfaction and job demands were mostly negative, but mixed findings existed for competence satisfaction. For example, some job demands (i.e., physical demands, stress, and conflict) were negatively related to competence satisfaction, whereas others were unrelated (i.e., workload and emotional demands) or even positively related (i.e., job demands).

**Table 4:** Interpreted within-person associations between job demands/stressors and daily or weekly basic psychological needs.

**Job demand/stressor**		**Article**	**Autonomy**	**Competence**	**Relatedness**	**Need satisfaction**
**Daily**						
1	Job demands	15	(Small^*^)	Small	(Small)	–
2	Emotional demands	6	(Small^*^)	(Small)	–	–
3	Physical demands	6	(Small^*^)	(Small^*^)	–	–
4	Cognitive demands	16	–	–	–	(Small)
5	Stress exposure	1	–	(Small^*^) to Medium^*^)	(Small^*^)	–
6	Work pressure	16	–	–	–	(Medium^*^)
7	Workload	6	(Small^*^)	Small	–	–
8	Family-work/work-family conflict	11	(Medium^*^)	(Medium^*^)	(Small^*^) to Medium^*^)	–
9	Time spent on core tasks	2	–	–	–	Small^*^
10	Time spent on administration	2	–	–	–	(Small)
11	Time spent on client interactions	2	–	–	–	Small^*^
12	Time spent on meetings	2	–	–	–	Small^*^
**Weekly**						
1	Job insecurity	4	–	–	–	(Medium^*^)
2	Job demands	13	(Small^*^) to Small	Small to Medium^*^	(Small^*^) to Small	–
3	Time spent working	20	(Small)	–	–	–
4	Regular work	20	Small^*^	–	–	–
5	Supplemental work	20	(Small)	–	–	
6	Progress	20	(Small)	–	–	–
**7**	**Unfinished tasks**	20,21	(Small)	(Small^*^)	–	–

*, *statistically significant; –, variables not measured; effect sizes, small (±0.1 to ±0.29); medium (±0.30 to ±0.49); large (±0.50 to ±1.00); brackets, negative associations; bolded variable, measured before basic psychological needs. The Article column refers to the study from which the finding was derived. Refer to Table 2 to identify the study referred to*.

Table 5 summarises an interpretation of the effect sizes of the within-person correlations between job resources and basic psychological need satisfaction from five studies. All job resources were measured concurrently with the needs. The job resources examined in the literature include skill utilisation, positive feedback, work and family aspects, job autonomy, and time spent on breaks. Job resources were positively related to variations in psychological need satisfaction, with a small to medium effect. There were some exceptions for the individual needs. For example, skill utilisation did not seem to affect daily autonomy and competence satisfaction. Job autonomy also did not have a significant relationship with weekly relatedness satisfaction.

**Table 5:** Interpreted within-person associations between job resources and daily or weekly basic psychological needs.

**Job resource**		**Article**	**Autonomy**	**Competence**	**Relatedness**	**Need satisfaction**
**Daily**						
1	Skill utilisation	6	Small	Small	–	–
2	Positive feedback	6, 19	Small^*^	Small to Small^*^	Small^*^	–
3	Family-work/work-family enrichment	11	Medium^*^	Small^*^ to Medium^*^	Small^*^	–
4	Work-life balance	11	Medium^*^	Medium^*^	Small^*^	–
5	Time spent on colleague interactions	2	–	–	–	Small^*^
6	Time spent on breaks	2	–	–	–	Small^*^
**Weekly**						
1	Job autonomy	13	Medium^*^	Small^*^ to Medium^*^	(Small) to Small	–

*, *statistically significant; –, variables not measured; effect sizes, small (±0.1 to ±0.29); medium (±0.30 to ±0.49); large (±0.50 to ±1.00); brackets, negative associations. The Article column refers to the study from which the finding was derived. Refer to Table 2 to identify the study referred to*.

Table 6 summarises an interpretation of the effect sizes of the within-person correlations between organisational resources and basic psychological need satisfaction from four studies. The organisational resources were measured concurrently with the needs. All the organisational resources in the diary studies were related to leader(ship) behaviours (or styles), which included transformational leadership and autonomy support vs. control. Organisational resources (in the form of supportive leader behaviours) were positively related to need satisfaction, mostly with a medium effect (four studies). A more controlling style was negatively associated with the need satisfaction, with a small effect (one study). Leaders(ship) behaviours always affected autonomy and relatedness satisfaction, while competence satisfaction was sometimes affected.

**Table 6:** Interpreted within-person associations between organisational resources and daily or weekly basic psychological needs.

**Organisational resource**		**Article**	**Autonomy**	**Competence**	**Relatedness**	**Need satisfaction**
**Daily**						
1	Autonomy support	11	Medium^*^	Medium^*^	Medium^*^	–
2	Transformational leadership	12	–	–	–	Small^*^
**Weekly**						
1	Autonomy-supportive communication	9, 10	Medium^*^ to Large^*^	(Small)	Medium^*^	–
2	Controlling communication	10	(Small^*^)	(Small^*^)	(Small^*^)	–

*, *statistically significant; –, variables not measured; effect sizes, small (±0.1 to ±0.29); medium (±0.30 to ±0.49); large (±0.50 to ±1.00); brackets, negative associations. The Article column refers to the study from which the finding was derived. Refer to Table 2 to identify the study referred to*.

Based on the findings in Tables 4–6, the significant daily and weekly within-person correlations between the work environment and need satisfaction ranged from a small to a large effect. Therefore, it can be concluded that variations in job demands, job resources, and organisational resources are associated with variations in psychological need satisfaction, as a whole or with specific needs (Review Objective 2a).

#### Employee Factors

Table 7 summarises an interpretation of the effect sizes of the within-person correlations between proactive employee behaviours/strivings and basic psychological need satisfaction from six studies. Proactive employee behaviours/strivings included proactiveness, job and leisure crafting, strivings, helping, enacted power, and performance. These behaviours/strivings were categorised as antecedents as some variables were measured before the basic psychological needs (striving behaviours, helping, and task performance). In contrast, the rest were measured concurrently (see Table 7). In the concurrent measurements (job/leisure crafting and proactive work behaviour), it was decided to include the behaviours as antecedents hypothesised in the included articles. Generally, fluctuating proactive employee behaviours/strivings correlated positively with fluctuating psychological need satisfaction, mostly with a small effect. However, there were some exceptions. Daily job crafting to reduce demands was not significantly related to need satisfaction, while weekly proactive behaviour did not relate to competence satisfaction.

**Table 7:** Interpreted within-person associations between proactive employee behaviours/strivings and daily or weekly basic psychological needs.

**Proactive employee behaviour/striving**		**Article**	**Autonomy**	**Competence**	**Relatedness**	**Need satisfaction**
**Daily**						
1	Daily job crafting to enhance social resources	3	Small^*^	Small^*^	Small^*^	–
2	Daily job crafting to enhance structural resources	3	Medium^*^	Medium^*^	Small^*^	–
3	Daily job crafting to reduce demands	3	Small	(Small)	(Small)	–
4	**Communion striving**	7	–	–	–	Small^*^
5	**Accomplishment striving**	7	–	–	–	Small^*^
6	**Status striving**	7	–	–	–	Small^*^
7	**Helping**	7	–	–	–	Small^*^
8	Enacted power	7	–	–	–	Small^*^
9	Proactive work behaviour	5	–	–	Medium^*^	–
10	**Task performance (engaging in achievement events)**	7, 19	Small^*^	Medium^*^	Small^*^	Small^*^
**Weekly**						
1	Leisure crafting	13	Small to Medium^*^	Small to Small^*^	Small to Medium^*^	–
2	Proactive work behaviour	21	–	Small	–	–

*, *statistically significant; –, variables not measured; effect sizes, small (±0.1 to ±0.29); medium (±0.30 to ±0.49); large (±0.50 to ±1.00); brackets, negative associations; bolded variables, measured before basic psychological needs. The Article column refers to the study from which the finding was derived. Refer to Table 2 to identify the study referred to*.

Table 8 summarises an interpretation of the effect sizes of the within-person correlations between pre-work employee states (i.e., attitudes or well-being experienced in the morning before going to work) and basic psychological need satisfaction from four studies. These pre-work states were all measured before need satisfaction (i.e., antecedents). The positive attitudinal or well-being factors included energy and positive affect (measured in the morning before work), while the negative factors were fatigue, anxiety, and negative affect before work. Employees' daily experiences of well-being or positive attitudes before work were positively related to variations in need satisfaction, with a small to medium effect. The negative dimensions were all unrelated to need satisfaction, as evident in three studies.

**Table 8:** Interpreted within-person associations between pre-work states (well-being/attitudes) and daily basic psychological needs.

**Pre-work state**		**Article**	**Autonomy**	**Competence**	**Relatedness**	**Need satisfaction**
**Positive factors**						
**1**	**Work engagement/ energy (before work)**	5, 14, 16	Medium^*^	Small to Medium^*^	Small^*^	Small^*^
**2**	**Positive affect (before work)**	7	–	–	–	Small^*^
**Negative factors**						
**1**	**Fatigue (before work)**	16	–	–	–	(Small)
**2**	**Anxiety (before work)**	5	–	(Small)	–	–
**3**.	**Negative affect (before work)**	7	–	–	–	(Small)

*, *statistically significant; –, variables not measured; effect sizes, small (±0.1 to ±0.29); medium (±0.30 to ±0.49); large (±0.50 to ±1.00); brackets, negative associations; bolded variables, measured before basic psychological needs. The Article column refers to the study from which the finding was derived. Refer to Table 2 to identify the study referred to*.

Table 9 summarises an interpretation of the effect sizes of the within-person correlations between employee demographic characteristics and basic psychological need satisfaction from two studies. Demographic characteristics were related to daily need satisfaction, with a small effect. Gender was negatively related to competence satisfaction (in a sample with 39% females) and positively to general need satisfaction (in a sample with 50.8% females). Age only related negatively to competence satisfaction. Both characteristics were unrelated to the other two needs. The number of children an employee had negatively related to their autonomy satisfaction but positively related to their competence and relatedness satisfaction. Employees' relationship status was negatively associated with their autonomy satisfaction but positively with their relatedness satisfaction.

**Table 9:** Interpreted within-person associations between demographic characteristics and daily basic psychological needs.

**Demographic characteristic**	**Article**	**Autonomy**	**Competence**	**Relatedness**	**Need satisfaction**
1	Gender	15, 16	(a)	(a)	(a)	(b)
			(Small)	(Small^*^)	Small	(Small^*^)
2	Age	15, 16	(a) Small	(a) (Small^*^)	(a) (Small)	(b) Small
3	Number of children (Mean: 0.91)	15	(Small^*^)	Small^*^	Small^*^	–
4	Relationship status (75% partner)	15	(Small^*^)	(Small)	Small^*^	–

*, *statistically significant; –, variables not measured; effect sizes, small (±0.1 to ±0.29); medium (±0.30 to ±0.49); large (±0.50 to ±1.00); brackets, negative associations. Gender, a. 39% female; b. 50.8% female. Age, a. Mean age was 42.61; b. Mean age was 34.90. The Article column refers to the study from which the finding was derived. Refer to Table 2 to identify the study referred to*.

Based on the findings in Tables 7–9, the significant daily and weekly within-person correlations between employee factors and need satisfaction ranged from a small to medium effect. Therefore, it can be concluded that employee demographic characteristics, fluctuating proactive behaviours/strivings, and pre-work employee states (i.e., attitudes and well-being before going to work) are associated with varying need satisfaction (Review Objective 2b).

### Relations Between Fluctuating Basic Psychological Need Satisfaction and Its “Outcomes”

#### Employee Attitudes and Well-Being

Table 10 summarises an interpretation of the effect sizes of the within-person correlations between basic psychological need satisfaction and employee outcomes (i.e., attitudes and well-being) from 17 studies. Most of the outcomes and the needs were measured concurrently, except for work engagement, striving behaviours, and psychological detachment. Attitudes and well-being were split into positive (e.g., work engagement, striving, detachment, self-control, positive affect, and relaxation) and negative (e.g., negative affect, burnout, stress, anxiety, and rumination) components. On a daily level, significant within-person correlations from 12 studies related positively to positive components and ranged from a small to large effect. On the other hand, these correlations were negatively related to negative components in eight studies, ranging from small to medium effect. On a weekly level, within-person correlations ranged from a small to large effect for both the positive (three studies) and negative (three studies) components of well-being and attitudes. Fluctuating need satisfaction was therefore associated with both positive and negative employee outcomes, varying from small to large effects. There were some exceptions. Autonomy satisfaction was related to all the employee outcomes, except for relaxation on a weekly basis. Similarly, competence and relatedness satisfaction are also related to all employee outcomes, except stress (not related to competence satisfaction daily or weekly), happiness (competence satisfaction), and negative affect (relatedness satisfaction).

**Table 10:** Interpreted within-person associations between employee outcomes (i.e., well-being and job attitudes) and daily or weekly basic psychological needs.

**Employee outcome**		**Article**	**Autonomy**	**Competence**	**Relatedness**	**Need satisfaction**
**Daily**						
1	Work enthusiasm/happiness	1, 2	–	Large^*^	Small^*^	Large^*^
**2**	**Work engagement**	3, 5, 11, 12, 14, 15, 16, 19	Small^*^ to Medium^*^	Small^*^ to Large^*^	Small^*^ to Large^*^	Medium to Large^*^
**3**	**Next-morning communion striving**	7	–	–	–	Small^*^
**4**	**Next-morning status striving**	7	–	–	–	Small^*^
**5**	**Psychological detachment from work**	5	–	Small^*^	–	–
6	Self-control	16	–	–	–	(Small^*^) to (Medium^*^)
7	Positive affect	7, 18	Small^*^ to Medium^*^	Small^*^ to Medium^*^	Small^*^	–
8	Negative affect	18	(Small^*^)	(Small^*^)	Small	–
9	Burnout	1, 3, 11, 16	(Small^*^) to (Medium^*^)	(Small^*^) to (Medium^*^)	(Small^*^)	(Small^*^)
10	Anxiety	5, 15	(Medium^*^)	(Small^*^)	(Small^*^) to (Medium^*^)	-
11	Strain/stress	6	(Small^*^)	Small	–	–
**Weekly**						
1	Happiness	10	Small^*^	(Small)	Medium^*^	–
2	Prosocial voice	4	–	–	–	Large^*^
3	Detachment from work	20	Small^*^	–	–	–
4	Mastery experiences	20	Small^*^	–	–	–
5	Relaxation	20	Small	–	–	–
6	Stress	10	(Small^*^)	Small	(Medium^*^)	–
7	Silence	4	–	–	–	(Medium^*^)
8	Negative affect	4	–	–	–	(Large^*^)
9	Affective rumination	21	(Medium^*^)	–	–	–

*, *statistically significant; –, variables not measured; effect sizes, small (±0.1 to ±0.29); medium (±0.30 to ±0.49); large (±0.50 to ±1.00); brackets, negative associations; bolded variables, measured after basic psychological needs. The Article column refers to the study from which the finding was derived. Refer to Table 2 to identify the study referred to*.

#### Employee Behaviours and Motivation

Table 11 summarises an interpretation of the effect sizes of the within-person correlations between basic psychological need satisfaction and performance/job-related outcomes (i.e., behaviours and motivation) from seven studies. All the outcomes were measured concurrently, except for striving behaviour. Performance/job-related outcomes included performance, striving, co-operation, oppositional defiance, irritation, and intrinsic motivation. On a daily level, significant within-person correlations from five studies related positively to the performance/job-related outcomes, ranging from a small to large effect. Weekly, within-person correlations of two studies ranged from a mostly small to medium effect. However, there were some exceptions. Need satisfaction was unrelated to accomplishment striving. Autonomy and relatedness satisfaction related positively to performance on a daily level, but not weekly. Competence satisfaction did not relate to co-operation and irritation with management. Finally, relatedness was unrelated to oppositional defiance toward instructions.

**Table 11:** Interpreted within-person associations between performance/job-related outcomes (i.e., job behaviours and motivation) and daily or weekly basic psychological needs.

**Performance/job-related outcome**		**Article**	**Autonomy**	**Competence**	**Relatedness**	**Need satisfaction**
**Daily**						
1	Performance	6	Small^*^	Medium^*^	–	–
**2**	**Next-morning accomplishment striving**	7	–	–	–	Small
3	Intrinsic motivation	16, 17, 18	Small to Large^*^	Small to Large^*^	Small to Small^*^	Medium^*^ to Large^*^
**Weekly**						
1	Performance	10	Small	(Small)	Medium^*^	–
2	Co-operation with management	9, 10	Small to Small^*^	Small	Medium^*^	–
3	Oppositional defiance toward instructions	9, 10	Small^*^ to Medium^*^	(Small^*^)	(Small)	–
4	Irritation toward management	9, 10	(Small) to Small^*^	(Small)	(Small^*^)	–
5	Intrinsic motivation	9, 10	Medium^*^ to Large^*^	Small^*^	Medium^*^	–

*, *statistically significant; –, variables not measured; effect sizes, small (±0.1 to ±0.29); medium (±0.30 to ±0.49); large (±0.50 to ±1.00); brackets, negative associations; bolded variables, measured before basic psychological needs. The Article column refers to the study from which the finding was derived. Refer to Table 2 to identify the study referred to*.

Based on the findings in Tables 10, 11, the significant daily and weekly within-person correlations between need satisfaction and employee outcomes ranged from a small to large effect. Therefore, needs satisfaction fluctuations are associated with employee outcomes, ranging from a small to large effect (Review Objective 3).

## Discussion

The objectives of this review were to evaluate if basic psychological need satisfaction varies on a daily and weekly level, and to judge on which level variations seem stronger, and to examine the relations between varying need satisfaction and its assumed antecedents and outcomes.

### Variations in Basic Psychological Need Satisfaction

The results indicated that need satisfaction varied intra-individually. Variations at the daily level were large (the medians ranging between 54.00 and 56.40%; Average ME = 55.60%) (Podsakoff et al., 2019) and seemed somewhat larger than at the weekly level (the medians ranging between 39.00 and 47.00%; Average ME = 42.41%). Notably, on a daily level, within-person variations accounted for somewhat more fluctuations in need satisfaction than between-person variations, whereas the opposite was true on a weekly level. Although more data is necessary to test this formally, it may prudently be suggested that the needs were more variable on a daily level, with the variability levelling off when moving to a weekly level.

Theoretically, employees may experience more fluctuations in their daily need satisfaction because their work environment fluctuates more on a daily level (Xanthopoulou et al., 2009, 2012; Bakker and Bal, 2010; Simbula, 2010). Employees may have “off days” or experience some days as more demanding or stressful than others (Simbula, 2010). Individual experiences (such as need satisfaction) may also fluctuate more on a daily basis due to the state-like nature of these constructs. Podsakoff et al. (2019) found that within-person variability often depended on the constructs being measured.

Methodologically, need satisfaction may fluctuate more on a daily level due to recall bias. Diary studies focus on events as they naturally occur, meaning that researchers do not have to depend on participants' recall bias (or remembered selves) (Bolger et al., 2003; Sonnentag and Geurts, 2009; Ohly et al., 2010; Kahneman, 2011), but rather on their experienced selves (see Kahneman, 2011). This bias is reduced when data is collected close to the event (Ohly et al., 2010) because individuals access their episodic memories shortly after an event. However, the larger the interval between the event and the recall, the greater the chances of individuals relying on their semantic memory. Semantic memory is more concerned with generalised beliefs (Lischetzke, 2014). More research is needed to control for the impact of measurement artefacts and to draw definite conclusions regarding the causes of greater fluctuations on a daily compared to a weekly level. According to Podsakoff et al. (2019), the number of days surveyed may not impact within-person variations, but the number of measurement points did matter. Diary studies typically include more measurement points than weekly studies, which could pose methodological challenges when comparing daily vs. weekly variations.

### Relations Between “Antecedents” and Fluctuating Basic Psychological Need Satisfaction

The results, furthermore, indicated that environmental and employee factors were related to employees experiencing more (or less) need satisfaction on some days (or in some weeks) than on/in others. In general, job demands were detrimental not only to overall need satisfaction but also to autonomy and relatedness satisfaction. Thus, on days or in weeks where employees had more job demands, they also experienced less need satisfaction. However, mixed findings existed for competence satisfaction. These findings are mostly in line with the findings from between-person studies (see Van den Broeck et al., 2016). Yet, there are some differences. For example, job demands related negatively to the three needs cross-sectionally (Van den Broeck et al., 2016), but only related negatively to daily and weekly autonomy and weekly relatedness. Several aspects may influence whether a demand is detrimental to need satisfaction. Firstly, employees' perception of a demand as a challenge or a hindrance determines whether it will relate positively or negatively to need satisfaction (Van den Broeck et al., 2010; Albrecht, 2015). Variability in how employees perceive or appraise demands (i.e., hindering or challenging) can also result in weak or non-significant associations between job demands and need satisfaction. These appraisals could explain additional variance in the effects of job demands on employee outcomes. Secondly, the level of the demand may play a role, with lower levels of demands (especially hindering demands) being more beneficial (i.e., curvilinear relationship) (Van den Broeck et al., 2010). Thirdly, the type of need measured may also play a role. For example, some job demands were negatively related to competence satisfaction, whereas others were unrelated or even positively related. More research is needed to understand the reasons for the differential relations between demands and need satisfaction.

In general, job and organisational resources were beneficial for need satisfaction. This finding aligns with the findings from between-person studies (see Van den Broeck et al., 2016). Thus, on days or in weeks where employees had more job and organisational (i.e., in the form of supportive behaviours) resources, they also experienced more need satisfaction. However, some of the relationships with job and organisational resources were unrelated (e.g., skill utilisation with daily autotomy and competence satisfaction and autonomy-supportive communication with weekly competence satisfaction). These findings are not surprising, as researchers argue that job (or organisational) resources are not equally beneficial; some resources may be more beneficial than others (Van Veldhoven et al., 2020). The reason for this is that the effects of job resources depend on the (a) nature and amount of the resource (i.e., how much value is attached to that resource), (b) individual context (i.e., personal resources, work behaviours, and attitudes of employees), (c) micro-context (i.e., level of job demands and other resources), (d) meso-context (i.e., organisation, supervisor, and employment practises), and (e) macro-context (i.e., country or culture) (Van Veldhoven et al., 2020). Future research should delve into this and examine when and why particular resources may be more beneficial than others.

In terms of employee factors, it was found that behaviours/strivings (engaged in to enhance work), attitudes, and well-being (as experienced before work) related positively to need satisfaction at work. Negative attitudes and ill-being (experienced in the morning before going to work) were unrelated. Need frustration refers to an active thwarting of psychological needs, which hinders psychological growth (Ryan and Deci, 2019), and should, thus, not be regarded as merely low need satisfaction (Vansteenkiste et al., 2020). These findings are mostly in line with between-person studies (see Van den Broeck et al., 2016). Thus, on days where employees experienced positive affect and energy in the morning before work, they also experienced more need satisfaction at work. However, their work-related need satisfaction remained unchanged when they experienced negative affect, anxiety, or fatigue before work. The non-significant relations with negative attitudes and ill-being before work might mean that other factors (in the work environment) helped to satisfy the needs, even when the employee was experiencing negative attitudes and ill-being before work. The non-significant relations could also indicate that negative attitudes and ill-being before work might rather be related to need frustration than to (low) need satisfaction. Similar to cross-sectional findings (see Van den Broeck et al., 2016), associations with demographic variables produced mixed results. Regardless of the direction of the relationship, employees' demographic characteristics only played a minor role in their daily experience of need satisfaction. This might be because demographic characteristics are more trait-like and often do not change.

Job and organisational resources and well-being and positive attitudes (before work) mattered more for need satisfaction than negative antecedents (e.g., job demands). This was even more true for organisational resources (such as supportive leader behaviours) because they mattered the most. Employees' behaviours showed the weakest associations with need satisfaction. So, providing employees with resources (especially supportive leaders) and facilitating their well-being and a positive attitude will have better outcomes for daily or weekly need satisfaction than focusing either on minimising job demands or addressing employee behaviours. Compared to the meta-analytic cross-sectional findings (see Van den Broeck et al., 2016), these findings differ somewhat. The meta-analytic confidence intervals (CIs) between job demands, job resources, and organisational resources overlapped, thus indicating that demands, resources, and factors in the organisational context were equally important for need satisfaction. Meta-analytic research focusing on diary studies is, therefore, needed to draw definite conclusions. However, from the literature, it is evident that the interpersonal behaviours of leaders are important for need satisfaction (Ryan and Deci, 2017; Slemp et al., 2018).

### Relations Between Fluctuating Basic Psychological Need Satisfaction and Its “Outcomes”

Finally, the results indicated that fluctuating basic psychological need satisfaction are associated with employee and performance/job-related outcomes. Need satisfaction is generally related positively to positive employee attitudes and well-being and performance outcomes, and negatively to negative employee attitudes and ill-being. Thus, on days or in weeks where employees experienced need satisfaction, they also experienced positive attitudes and well-being and were more likely to be motivated. Hence, they displayed more positive job behaviours and fewer negative job behaviours when their needs were satisfied. The findings are consistent with between-person studies (see Van den Broeck et al., 2016). However, some of the relationships were unrelated (e.g., relatedness satisfaction with negative affect, competence satisfaction with happiness, relatedness satisfaction and oppositional defiance toward instructions). Again, different needs (i.e., autonomy, competence, and relatedness satisfaction) associated differently with different outcomes.

It appeared that need satisfaction mattered more for employee attitudes and well-being than performance, but the most for intrinsic motivation. This differs from cross-sectional meta-analytic findings (see Van den Broeck et al., 2016) that showed that the CIs between attitudes, well-being, and performance overlapped, meaning that need satisfaction mattered equally for these outcomes. It is, however, plausible that other factors such as ability might have a bigger influence on performance (Van Iddekinge et al., 2014) than need satisfaction. However, evidence from cross-sectional between- and within-person studies repeatedly showed that the basic psychological needs were essential for motivation, well-being, and performance (Vansteenkiste et al., 2020).

Notably, this review shows the vast diversity of work-related and personal antecedents of need satisfaction that has been studied in diary studies and the various outcomes of within-person fluctuations of need satisfaction. The majority of the diary research echoes research that has been conducted at the between-level, yet both streams of the literature also show some complementarity. Diary studies could further examine factors that have been tapped into in between-person research, such as job insecurity, social support from colleagues and/or counter-productive behaviour, but should particularly focus on capturing the dynamics that typically happen across experiences. For instance: How do breaks in the morning co-vary with need satisfaction in the afternoon or would need satisfaction in the morning influence helping behaviour later that day? Diary studies could also examine personality variables, as Van den Broeck et al. (2016) included in their meta-analysis (e.g., Big Five personality traits). Podsakoff et al. (2019) explained that factors (such as personality) that have traditionally been studied as stable between-person variables could show meaningful within-person variation when paired with certain antecedents.

In conclusion, daily (and, to some extent, weekly) need satisfaction matters in the workplace due to its relations with well-being and performance. However, needs seem to be more dynamic on a daily level, while the variability of needs tapers off when one measures it on a weekly level. Furthermore, the role of the daily variations in the work environment and the individual (in explaining variations in need satisfaction) and the role of daily variations in need satisfaction (in explaining variations in employee behaviour, well-being, and motivation) can not be ignored. Different “antecedents” and “outcomes” also associate differently with the different needs. Thus, one can draw greater insights by measuring the needs as separate constructs instead of unidimensional ones. Finally, need satisfaction is not irrelevant when studying negative outcomes, but measuring need frustration may be more valuable (Van den Broeck et al., 2016), especially in the context of antecedents. Although the general trends seem to be similar for diary and cross-sectional designs, much can still be learnt about need satisfaction if studied daily. As neither the experienced self nor the work environment is static, insights can be developed into employees' daily attitudes, behaviours, and well-being and how to manage these.

## Limitations and Recommendations for Future Research

One major limitation was the paucity of available research related to diary studies of basic psychological needs in the work context. In their meta-analysis, Van den Broeck et al. (2016) found 99 cross-sectional papers to review, whereas we had to draw conclusions based on 21 articles. Due to the limited number of diary studies, meta-analytic procedures were not possible. It should, furthermore, be noted that more daily studies than weekly studies were available for inclusion in this review, which may influence the conclusions drawn. This limitation makes it challenging to compare the results of between-person vs. within-person, and daily vs. weekly variations, as measures of central dispersion may be biassed. Also, one cannot employ commonly used techniques to test statistical significance, effect sizes, or confidence intervals.

Another limitation was that most of the diary studies in this review focused on need satisfaction only. Future diary studies should also include the dynamics of need frustration, as Vansteenkiste et al. (2020) mention that need satisfaction and frustration should be regarded as two-dimensional constructs with different antecedents and outcomes.

When designing diary studies, researchers must consider the impact of retrospective bias. Shorter periods (between the event and its measurement) may be more meaningful if the *experienced self* (Kahneman, 2011) is studied. Kahneman and Riis (2005) believe that evaluated well-being (i.e., recall and retrieval of the experience) differs from the actual experience (i.e., experienced well-being). Therefore, focusing on the *experienced self* instead of the *remembered self* could reduce retrospective bias. Focusing on diary studies and measuring a person's actual experiences (*experienced self*) is important, as relationships between constructs are sometimes different on the within-person compared to the between-person level (McCormick et al., 2018). Thus, how individuals vary from one another could be different from how they vary within themselves. Given the above and the finding that need satisfaction varies more daily, it is thus recommended that need satisfaction be studied as a daily variable.

Podsakoff et al. (2019) emphasise that the construct type, measurement, and design of the study and the sample characteristics could account for differences in within-person variances. Once more diary research on need satisfaction becomes available, a recommendation could be to conduct a meta-analysis on diary studies to control for these factors.

Some of the studies included did not separate the constructs in time (i.e., the “antecedents” and “outcomes” were measured concurrently). As a result, the authors had to use existing theoretical justifications to classify variables as antecedents or outcomes. The original articles' objectives or hypotheses were also consulted in this regard. Definite conclusions on whether the concurrent measurements were antecedents or outcomes could therefore not be made. It is recommended that researchers measure the constructs at different time points to draw definite conclusions regarding the directions of the relationships.

## Managerial Implications

Antecedents of need satisfaction, in the form of an employee's social context or environment (e.g., job resources, positive leadership), mattered more for varying need satisfaction than job demands and personal factors. The organisational context seemed to play the most crucial role in need satisfaction. Thus, interventions focused on the organisational context could be suggested. Organisations should consider prioritising organisational resources (e.g., leadership development to enhance leaders' need-supportive behaviours and organisational redesign and reformulation of policies and procedures) followed by job resources (e.g., ensuring social support and skill utilisation and providing positive feedback), instead of merely trying to reduce job demands (e.g., reducing stressors) or teaching employees to manage the demands. Specifically, based on our results, organisations should be aware that need satisfaction can vary considerably from day to day and—in response—should make sure that employees have access to job and organisational resources (e.g., task autonomy, social support, positive leadership, etc.) on a daily basis. For example, managers can have short daily check-ins with team members (if required) to understand their daily resource or demand experiences. This will enable management to keep track and facilitate employees' need satisfaction experiences. They can also ask team members to inform them if they have problems accessing certain resources or managing certain demands. The findings of this study might suggest that more hands-on leadership could be beneficial for need satisfaction.

## Data Availability Statement

The original contributions presented and used in the study are available from the corresponding author on request.

## Author Contributions

LC acted as the primary researcher as this study forms part of her doctoral research. LvdV, AV, and SR acted as supervisors. They played an advisory role, assisting in the conceptualisation of the study, assisting with the interpretation of the research results, and refining the manuscript. All authors contributed to the article and approved the submitted version.

## Funding

The study was funded by the National Research Foundation (TTK190307422577), but the ideas and opinions remain those of the researchers.

## Conflict of Interest

The authors declare that the research was conducted in the absence of any commercial or financial relationships that could be construed as a potential conflict of interest.

## Publisher's Note

All claims expressed in this article are solely those of the authors and do not necessarily represent those of their affiliated organizations, or those of the publisher, the editors and the reviewers. Any product that may be evaluated in this article, or claim that may be made by its manufacturer, is not guaranteed or endorsed by the publisher.
